# Evidence of a Double-Lid Movement in *Pseudomonas aeruginosa* Lipase: Insights from Molecular Dynamics Simulations

**DOI:** 10.1371/journal.pcbi.0010028

**Published:** 2005-08-12

**Authors:** Subbulakshmi Latha Cherukuvada, Aswin Sai Narain Seshasayee, Krishnan Raghunathan, Sharmila Anishetty, Gautam Pennathur

**Affiliations:** 1 Centre for Biotechnology, Anna University, Chennai, India; 2 AU-KBC Research Centre, Madras Institute of Technology, Chennai, India; Cornell University Weill Medical College, United States of America

## Abstract

*Pseudomonas aeruginosa* lipase is a 29-kDa protein that, following the determination of its crystal structure, was postulated to have a lid that stretched between residues 125 and 148. In this paper, using molecular dynamics simulations, we propose that there exists, in addition to the above-mentioned lid, a novel second lid in this lipase. We further show that the second lid, covering residues 210–222, acts as a triggering lid for the movement of the first. We also investigate the role of hydrophobicity in the movement of the lids and show that two residues, Phe214 and Ala217, play important roles in lid movement. To our knowledge, this is the first time that a double-lid movement of the type described in our manuscript has been presented to the scientific community. This work also elucidates the interplay of hydrophobic interactions in the dynamics, and hence the function, of an enzyme.

## Synopsis

Lipases hydrolyse long-chain fatty acid esters at water-oil interfaces through the mechanism of interfacial activation mediated by the movement of a lid subdomain that covers the active site. Studying lid movement is an area of active research in the field of protein dynamics. The lipase from *Pseudomonas aeruginosa* is a 29-kDa protein that was previously crystallized in the open conformation, and as expected, an approximately 20-residue lid subdomain was identified. In the present study, the authors report extensive molecular dynamics simulations of the *P. aeruginosa* lipase. They show that this protein has two lids covering the substrate-binding pocket. The first lid is the one proposed from the known crystal structure. The second lid, a much shorter one, lies over the binding pocket facing the first lid. Furthermore, using position-restrained simulations, these authors show that movement of the second lid may actually be a trigger for the movement of the first, and that this triggering action is driven by hydrophobic contacts between the two lids. This computational study paves a way for experimentalists to study the structure and dynamics of this protein in greater detail in order to understand coupled subdomain movements in a comprehensive fashion.

## Introduction

Lipases are enzymes that catalyze both the formation and cleavage of long-chain acylglycerols [1]. In recent years, crystal structures of several lipases—bacterial, fungal and mammalian—have been determined [2–8]. Industrially, lipases—especially those of bacterial and fungal origins—are seemingly attractive as biocatalysts due to their cofactor independence, broad substrate specificity, enantioselectivity, and stability in organic solvents [9]. In addition, there have been a number of reports implicating lipases in pathogenicity and biofilm formation [10,11].

All lipases, despite extensive sequence divergence, have a common fold known as the alpha/beta hydrolase fold [12]. The canonical alpha/beta hydrolase fold has eight parallel beta strands, with the second one anti-parallel. Strands 3–8 are connected by alpha helices, which pack on either side of the beta sheet. The active site of lipases is the classical catalytic triad composed of a serine, an aspartate/glutamate, and a histidine [13]. An interesting characteristic of lipases is their interfacial activation. One of the common modes by which this activation occurs is through a lid, also called flap: a stretch of amino acids that moves towards closing the hydrophobic binding pocket in an aqueous environment and opens in an oil-water interface [14]. For further details, see Protocol S1.

The lipase in this study—that from *Pseudomonas aeruginosa*—is a 29-kDa protein. This lipase differs from the canonical alpha/beta hydrolase fold in that the first two beta strands and one alpha helix are missing. The active site residues in this lipase are Ser82, Asp229, and His251. The loop containing the active-site histidine is stabilized by an octahedrally coordinated calcium ion. On top of the active site, a lid subdomain, comprising the alpha helix residues 125–148 and its surrounding loops, is present in an open conformation in the crystal structure [15].

Molecular dynamics (MD) studies have been used extensively in the study of protein folding [16] and enzyme catalysis [17]. MD simulations have been used to show that there are three mobile regions in the lipase from *Rhizomucor miehei,* one of which corresponds to the active-site lid [18]. The role of lids and protein orientation with respect to the lipid-water interface in lipase catalysis has been investigated using MD simulations [19,20]. Dependence of enantioselectivity of *Candida antarctica* lipase on temperature has also been studied using MD [21]. Our group has recently used MD simulations to explain pH-dependent enantioselectivity of *C. rugosa* lipase and has elucidated the role of the lid in the same [22]. A short introduction to MD simulations is provided in Protocols S2, S3, and S4.

Lipases were traditionally believed to have a single lid. By carrying out extensive molecular dynamics simulations, we infer the presence of a second lid in *P. aeruginosa* lipase. (The lid proposed from the crystal structure is henceforth called lid1, and the second lid as seen from our results, lid2.) We show that the movement of lid2 acts as a trigger for the movement of lid1. Using similar MD simulations of rational in-silico mutants of this lipase, we elucidate the importance of hydrophobic interactions in movement of the lids. Thus we provide novel insights into interfacial activation in *P. aeruginosa* lipase.

## Results/Discussion

Comparisons of the crystal structures of the open and the closed forms of lipases have been used to conclusively identify mobile loops. However, the crystal structure of only the open form of the lipase from *P. aeruginosa* is available. Under these circumstances, it is imperative to use other techniques, such as MD simulations, to identify such mobile loops that are essential for the enzyme's activity. Simulations of the open and the closed form of *Humicola lanuginose* lipase, a fungal lipase, in water for 12 ns using a standard procedure has shown that the open form tends to move towards the closed conformation, while not closing entirely, whereas the closed form remains as such [19]. Ours is, to our knowledge, the first comprehensive study on the dynamics of *P. aeruginosa* lipase.

The open structure of *P. aeruginosa* lipase was obtained from the Protein Data Bank (PDB). This was solvated in explicit water and energy minimized. The energy-minimized structure was subjected to MD simulation for 20 ns. The backbone root mean square deviation (RMSD) as calculated after least square fit increased steadily up to about 2.5 ns and reached a plateau. The RMSD value started increasing again after about 4.5 ns and stabilized at around 7 ns. General simulation parameters are shown in Protocol S5.

### The First Lid

**Figure 1:**
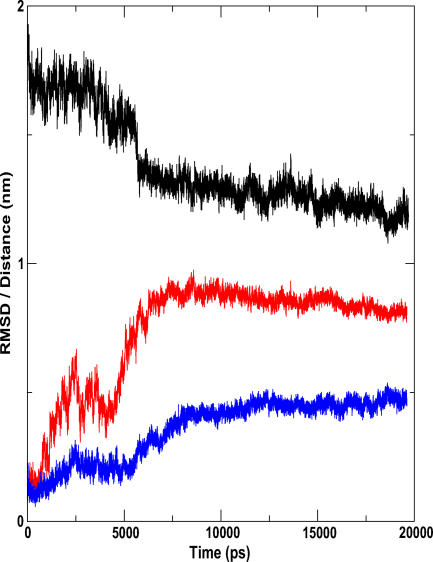
Lid Movement in *P. aeruginosa* Lipase: Quantitative Measures Here we show the time variation of the distance between the lids and the RMSD of the two flaps as a function of time. The RMSD of lid1 (red) after least-square fit of the protein backbones is approximately 0.9 nm. The RMSD of the helix in lid2 (blue) is also shown. This is corroborated by a plot of distance between the two lids against time (black). There is an appreciable decrease in this distance after 6 ns. The lid1-lid2 distance stabilizes at about 8 ns, after which there is little relative movement between the two lids.

Since the starting structure is in the open, active conformation, and lipases are known to be activated only at lipid-water interfaces, lid1 can be expected to close in aqueous environments. When the simulated structure (represented here by the 10-ns snapshot) is superimposed on the energy-minimized starting structure, and lid1 highlighted, the movement of this lid towards a typical closed conformation becomes apparent. It can also be seen that the mobile region consists of a helix-loop-helix motif. In the crystal structure, the first helix covers residues 113–117 while the second helix encompasses residues 126–146. In this helix-loop-helix motif, residues 116–138 show high RMS deviations. The C-terminal portion (139–146) of the second helix remains largely rigid. The loop region following this motif—residues 150–156—appears to be highly flexible. This lid has moved by a distance of 1.25 nm, as measured by the distance between the Ca atoms of V130 in the starting structure and in the simulated structure. The RMSD of the lid after least-square fit of the protein backbones is approximately 0.9 nm (Figure 1).

### The Second Lid

**Figure 2:**
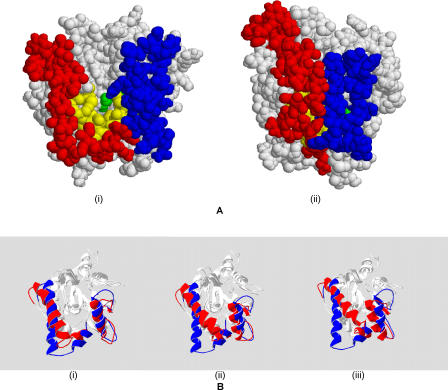
Lid Movement in *P. aeruginosa* Lipase: Qualitative Representation (A) This figure represents the simulation results. The structure on the left (i) is the open form of the lipase, which reveals the active site cavity. Binding pocket residues are coloured yellow, and lid residues red (lid1) and blue (lid2). There appears to be nearly a 100% decrease in the solvent-accessible area when the lids close (structure on right [ii]). (B) Time evolution of the structure backbone is shown. Blue corresponds to the open crystal structure of the lipase, while red indicates the position of the lids after 3 ns (left [i]), 7 ns (centre [ii]), and 10 ns (right [iii]).

On careful analysis we find two regions, besides lid1, that show high mobility. This includes a stretch of amino acids from residues 20 to 30 and another from 203 to 228. Of the two, the latter is found to move towards lid1, thereby augmenting the effect of the movement of lid1. While most residues in the 203–228 region belong to loops, residues 210–222 compose an alpha helix, whose movement towards the opposite lid is significant. When we refer to lid2, we point to the above-mentioned helical region. A spacefill model of both the starting and the simulated structures (Figure 2A) clearly reflects the effect of the movement of the lids in reducing the solvent accessibility of the binding pocket. Quantitatively, the exposed area of the side chains forming the pocket HA decreased from 98.4 Å^2^ to 0.86 Å^2^, which is almost a 100% decrease. This has led us to the conclusion that there is a second lid, which, together with the first lid, acts as a double door in protecting the hydrophobic binding site from the polar solvent. The structures of the lipase obtained at different time points of the simulation are shown in Figure 2B.

The 10 ns structure does not differ much from the final 20-ns structure (lid1 and lid2 RMSDs are less than 0.1 nm) and hence was taken as the representative structure on the basis of which simulations involving the closed form were performed. Also, the third highly mobile region, residues 20–30, does not cover the binding pocket and hence is not included in further discussion. This movement is touched upon in Protocol S6. Moreover, restraining the movement of this third loop does not seem to affect lid movement in any manner.

**Figure 3:**
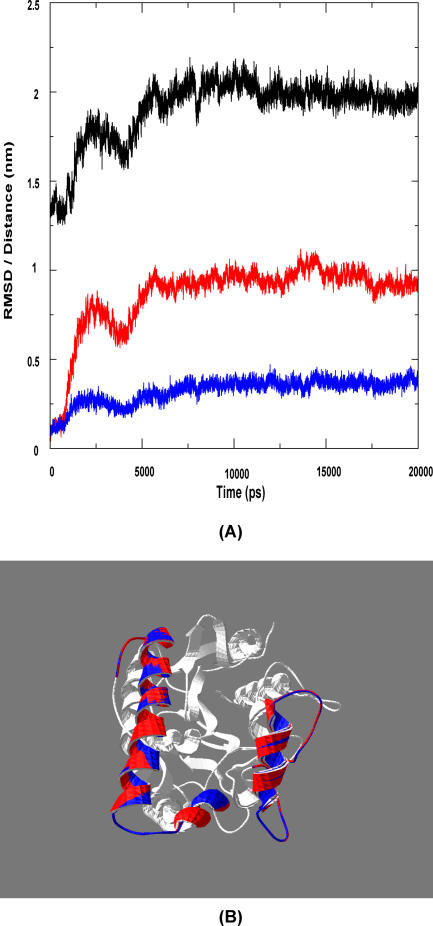
Simulation Results of Closed Form in Octane-Water Interface This model reiterates our hypothesis of the second lid in the lipase. (A) This shows the variation of the distance (black) and RMSD of the two lids (red for lid1 and blue for lid2) with the time course of simulation. As the lid opens, there is a corresponding increase in distance between the two lids. This movement stabilizes from 8 ns until the end of simulation. (B) Here the final structure of the simulation (after 20 ns; red) is superimposed on the crystal structure (blue) to a C-alpha RMSD of 0.08 nm.

A “back” simulation was performed using the closed structure in a water-octane interface. We observed that both the lids actually opened out, and the structure thus formed was nearly the same as the initial crystal structure. The RMSD variation of the lids during the course of the simulation is shown in Figure 3A. The open form obtained after 20 ns and the crystal structure are superimposed to a C-alpha RMSD of 0.08 nm and is shown in Figure 3B.

### Lid2 Movement Triggers Movement of Lid1

**Figure 4:**
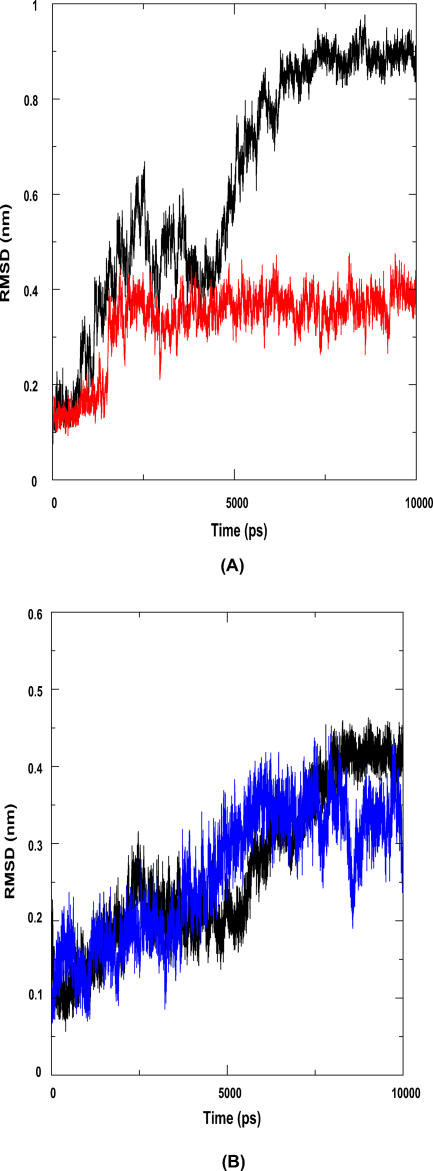
Lid1 Movement is Triggered by Lid2, but Lid2 Movement Is Independent of Lid1 (A) Red represents the movement of lid1 when lid2 is restrained, while black is that in the unrestrained system. The movement of lid1 is significantly hindered when lid2 is restrained. (B) Blue represents the movement of lid2 when lid1 is restrained, and black represents the movement in the unrestrained system. The movement of lid2 is the same in both cases.

In an earlier paper, Gunasekaran and coworkers [23] demonstrated the effect of triggering loops in enzyme catalysis using restrained MD. To investigate the interdependence of the movements of the two lids, we have applied the same approach. For this we carried out two separate simulations, one with lid1 restrained and the other with lid2 restrained. We observe that while each lid, with the other lid restrained, moves initially, subsequent and significant movement of lid1 is seen only when lid2 is also mobile. RMSD of lid1 increased to 0.4 nm in about 2 ns in both the unrestrained and lid2-restrained systems. The second RMSD increase at about 5 ns, from 0.4 to 0.9 nm, seen in the unrestrained system, is absent in the lid2-restrained system (Figure 4A). The extent of movement of lid2 appears to be independent of that of lid1. In both the unrestrained and the lid1-restrained systems, the RMSD of lid2 in the stable state is approximately 0.4 nm. However, the nature of the trajectory is different in the two cases. In the restrained case, lid2 moves to an RMSD of 0.4 nm within 6 ns, while this is achieved only after 8 ns in the case of the unrestrained molecule (Figure 4B). However, this minor difference can be attributed to the chaotic nature of MD simulations.

### Lid Closure is Driven by Hydrophobic Interactions

**Figure 5:**
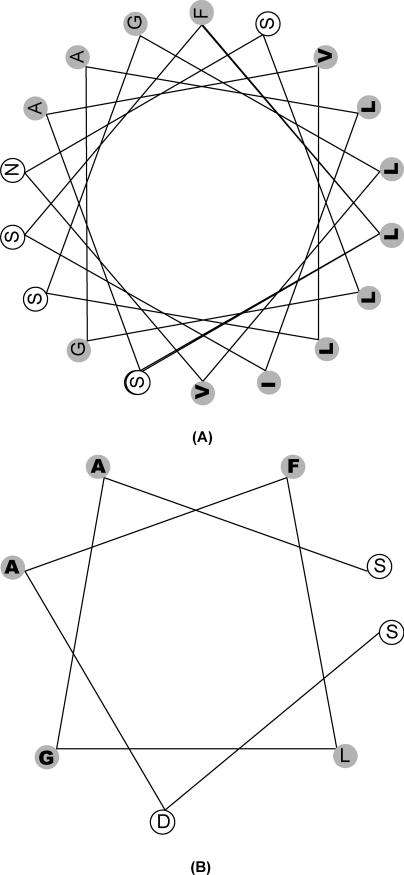
Helical Wheel Diagrams for Lid1 and Lid2 Shown are the long helix in lid1 (A) and the helix in lid2 (B). Hydrophobic residues are shaded in grey; those in bold face the binding pocket and opposite lid. Letters stand for amino acids.

A helical wheel representation of the two lids (Figure 5) in the *Pseudomonas* lipase shows that the helices involved are amphipathic, with hydrophobic side chains protruding towards the binding site and towards the opposite lid. The presence of this hydrophobic surface facing the binding site is an ideal construction that allows the binding cleft and the lids to come together through hydrophobic interactions. We hypothesize that significant movement of lid1 corresponding to the 5 ns region in the unrestrained system may require the presentation of hydrophobic residues by lid2 in near proximity to lid1, which does not happen when lid2 is restrained. In fact, close (less than 0.1 nm between contacting surfaces) contacts between Ala126, Val130, and Leu131 on lid1, and Pro210, Phe214, and Ala217 on lid2, are seen in the final stable structure obtained from the unrestrained simulation. This led us to look at the role of hydrophobic interactions in the lid movement. To get further insights into the movement of the lids, we performed further MD with rational in silico mutants.

### The Role of Hydrophobic Interactions in Interfacial Activation

We performed molecular dynamics on different in silico mutants of the two residues in lid2, Ala217 and Phe214, so as to implicate the role of hydrophobicity in the movement of the lids. These residues were chosen because they make contacts with residues on lid1 in the closed conformation. Pro210, which also contacts residues on lid1 in the closed conformation, was left untouched because of the possibility of profound local impacts that a proline can impart to a structure. This Pro acts as the N-terminal cap to the lid2 helix and may also be a hinge residue. The aim of this study is to assess the importance of hydrophobic residues in the triggering lid on the movement of the major lid1, and hence only residues from lid2 were mutated.

**Figure 6:**
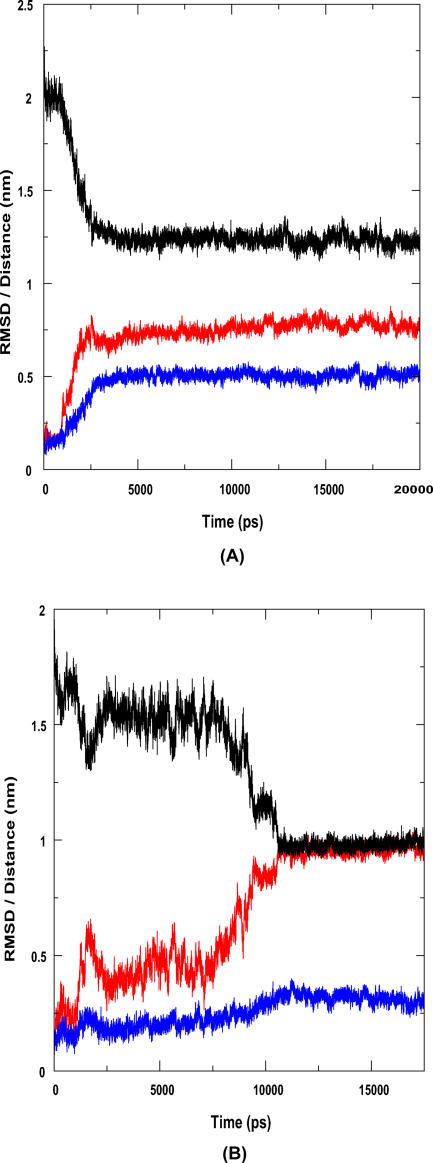
Simulations with Lipase Single Mutants (A) This figure corresponds to the simulation of mutant Ala217Ser. The movement of the lids very similar to those in the wild-type lipase. Thus, despite the mutation, the lids are able to close in an aqueous environment. (B) Here, the simulated structure is a mutant in Phe214Ser. Red, lid1 RMSD; blue, lid2 RMSD; black, lid1-lid2 distance.

At the first level, we carried out MD studies on single mutants: the Phe214 and Ala126 were independently mutated to Ser in two different simulations. This was done so as to investigate the role of individual residues in domain movement. These mutations do not seem to affect the lid movement, which is almost the same as in the wild type. The RMSDs of the lids are similar in both the cases (Figure 6; for qualitative information, see Protocol S7).

**Figure 7:**
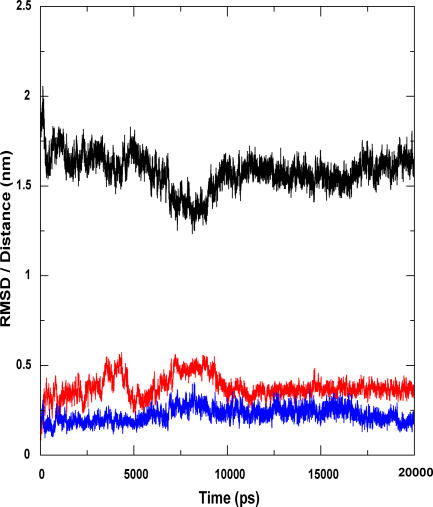
Simulation with Lipase Double Mutants Both the alanine at 217 and the phenylalanine at 214 were mutated to serines, and the simulation was carried out in an aqueous medium. The lids do not close in an aqueous environment. Thus, by mutating the key contact residues to serines, the hydrophobicity of lid2 is lost, and it is unable to function as a trigger for the lids to converge and close. Red, lid1 RMSD; blue, lid2 RMSD; black, lid1-lid2 distance.

Subsequently we performed a double mutation, Phe214Ser/Ala217Ser. After 20 ns of simulation, the lids did not close. The RMSD plot of the simulation is highly fluctuating and there is nearly no change in the distance between the lids (Figure 7). Thus the double mutation affected the lid movement to a great extent, establishing the robustness that multiple hydrophobic contacts confer to the function of a protein.

**Figure 8:**
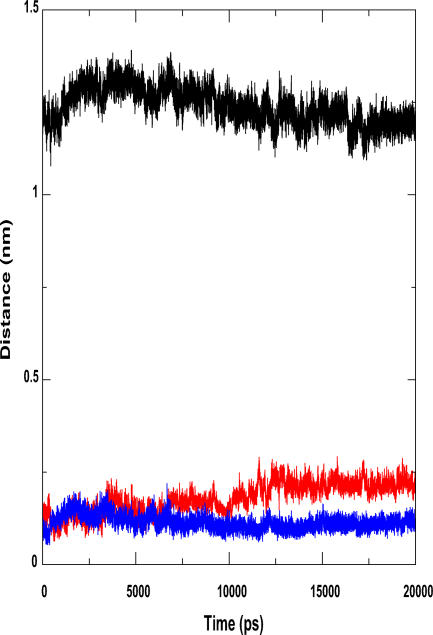
Simulation of Closed-Structure Double Mutant in Octane-Water Interface The closed-structure mutant Phe214Ser/Ala217Ser was simulated in an oil-water interface. The lids do not open to expose the lid to the octane-water interface. Thus, hydrophobicity appears to be essential for the lids to open. Hydrophobic affinity between the lid and the octane may be higher than that between the lids, allowing the lids to move apart. In the absence of such hydrophobic groups in lid2, this movement does not take place. Red, lid1 RMSD; blue, lid2 RMSD; black, lid1-lid2 distance.

We have also looked at the importance of hydrophobicity in maintaining the closed structure. The closed form of the lipase was mutated at both residues Phe214 and Ala217 to Ser. This structure was simulated in both water and an octane-water interface. In both cases, the lids failed to open. This behaviour in water, where the wild-type lipase is expected to be in a closed form, shows that these hydrophobic contacts are essential only to drive the lid-closure process, but they do not affect the stability of the closed conformation. That the mutant is unable to open up in a water-octane interface, contrary to the wild type (Figure 8), shows that hydrophobicity appears to be essential to lid opening and hence interfacial activation as well. Possibly there is a greater hydrophobic affinity between octane and lid2 than between the lids; thus there appears to be an intricate role for hydrophobicity in the domain movement of lipase.

### Conclusions and Future Directions

We have performed restrained and unrestrained MD simulations of *P. aeruginosa* lipase in an aqueous environment. This is a lipase whose structure has not been studied as extensively as fungal lipases, leading to paucity of data. We show that this lipase has two lids covering the binding cleft in its putative closed conformation, and that the movement of each lid is not entirely independent of that of the other. We also investigated the role played by an intricate hydrophobic interaction network in the dynamics and, hence, the function of the enzyme. While MD techniques, given the highly developed nature of the force fields used, are fairly accurate, the results that we show amount to a strong hypothesis. However, our theories are amenable to experimental verification. A crystal structure of the closed form of the lipase would allow comparison of experimentally determined structures of the two conformations of the lipase and hence identify mobile loops. On the other hand, fluorescence resonance energy transfer experiments, coupled with site-directed mutagenesis, could be carried out to elucidate the role of hydrophobic contacts in driving lid closure.

## Materials and Methods

### Software and infrastructure.

The simulations were carried out using GROMACS 3.2 running on single IBM 2.8 GHz systems loaded with Fedora Core 2 Linux at the BTIS facility, Centre for Biotechnology, Anna University. Visualizations were done on an SGI Fuel workstation. GROMACS [24,25] was downloaded from http://www.GROMACS.org.

### Simulation.

The GROMACS force field was used for the simulations. The calcium that stabilizes the active-site histidine-containing loop was treated using nonbonded potentials, and it was found that this loop is rigid. The protein was solvated in a box that left a space of 0.2 nm around the solute. SPC water molecules (approximately 3,000) were added. All nonbonded potentials were truncated at 1 nm. For energy minimization, the steepest descents method was used. During the MD, grid-type neighbour searching was done. Long range electrostatics was handled using PME [26]. The system was weakly coupled to an external bath using Berendsen's method [27]. The reference temperature was fixed at 300 K. All bonds were constrained with LINCS [28,29]. A time step of 2 fs was used. Structures were written every 0.5 ps to the trajectory. Each nanosecond took approximately 18 h of CPU time.

The above standard procedure has been used by Jensen et al. [19] to simulate the pair of open and closed forms of *H. lanuginose* lipase in aqueous conditions. They show that, in 12 ns, the open form tends to move towards a closed conformation, while the closed conformation remains as such.

In order to generate the water-octane interface, a water box (~3,000 water molecules), similar to the one used for the aqueous simulations, was built around the protein. A second box containing ~200 molecules of octane was laid over the water box. Following this, standard simulation protocols were used. The protein molecule, with its lid facing the octane box, moved to the interface within the first nanosecond. All box dimensions are given in Protocol S8.

### Analysis.

All visualizations were done using Rasmol or the Swiss PDB Viewer. Trajectory analyses were carried out using tools built inside the GROMACS package. RMSDs were calculated for the protein/protein fragment backbone using least squares fit. The radius of gyration was also calculated for the backbone. Solvent-accessible surface area [30] was calculated using Whatif [31] for the side chains of the residues making up the acyl-binding pocket (HA). Lid1-lid2 distance was calculated between the respective centres of mass. For computing hydrogen bonds, cut-off donor-acceptor distance was 0.35 nm.

## Supporting Information

Protocol S1 Lipases (154 KB PDF).

Protocol S2 MD Simulations: A Short Perspective (17 KB PDF)

Protocol S3 MD Simulations: A Model Protocol (97 KB PDF)

Protocol S4 MD Simulations: Position Restraints (14 KB PDF)

Protocol S5 Simulation Parameters (71 KB PDF)

Protocol S6 The Third Mobile Loop (97 KB PDF)

Protocol S7 Single Mutants (29 KB PDF)

Protocol S8 Box Sizes (7 KB PDF)

Video S1 Main Simulation Movie (2.1 MB WMV)

### Accession Numbers

The *P. aeruginosa* lipase PDB (http://www.rcsb.org/pdb/) accession number is 1EX9, and the Uniprot (http://www.ebi.ac.uk/swissprot/access.html) accession number is P26876.

## Abbreviations

MD: molecular dynamics
PDB: Protein Data Bank
RMSD: root mean square deviation

## References

[pcbi-0010028-b01] Jaeger KE, Dijkstra BW, Reetz MT (1999). Bacterial biocatalysis: Molecular biology, three-dimensional structures and biotechnological applications of lipases. Annu Rev Microbiol.

[pcbi-0010028-b02] Grochulski P, Li Y, Schrag JD, Bouthillier P, Smith P (1993). Insights into interfacial activation from an open structure of *Candida rugosa* lipase. J Biol Chem.

[pcbi-0010028-b03] Brady L, Brzozowski AM, Derewends ZS, Dodson G, Tolley S (1990). A serine protease triad forms the catalytic centre of a triacylglycerol lipase. Nature.

[pcbi-0010028-b04] Brzozowski AM, Savage H, Verma CS, Turkenburg JP, Lawson DM (2000). Structural origins of interfacial activation in *Thermomyces (Humicola) lanuginosa* lipase. Biochemistry.

[pcbi-0010028-b05] Schrag JD, Cygler M (1993). 1.8A refined structure of the lipase from *Geotrichum candidum*. J Mol Biol.

[pcbi-0010028-b06] Carriere F, Thirstrup K, Hjprth S, Ferrato F, Nielson PF (1997). Pancreatic lipase structure-function relationships by domain exchange. Biochemistry.

[pcbi-0010028-b07] Schrag JD, Li Y, Cygler M, Lang D, Burgdorf T (1997 ) The open conformation of a *Pseudomonas* lipase. Structure.

[pcbi-0010028-b08] Derewenda ZS, Derewenda U, Dodson CG (1992). The crystal and molecular structure of *Rhizomucor miehei* triacylglyceride lipase at 1.9 A resolutions. J Mol Biol.

[pcbi-0010028-b09] Jaeger KE, Reetz MT (1998). Microbial lipases form versatile tools for biotechnology. Trends Biotechnol.

[pcbi-0010028-b10] Berto P, Commenil P, Belingheri L, Dehorter B (1999). Occurrence of a lipase in spores of *Alternaria brassicicola* with a crucial role in the infection of cauliflower leaves. FEMS Microbiol Lett.

[pcbi-0010028-b11] Stehr F, Kretschmar M, Kroger C, Hube B, Schafer W (2003). Microbial lipases as virulence factors. J Mol Catalysis.

[pcbi-0010028-b12] Schrag JD, Cygler M (1997). Lipases and alpha/beta hydrolase fold. Methods Enzymol.

[pcbi-0010028-b13] Ollis DL, Cheah E, Cygler M, Dijkstra B, Frolow F (1992). The alpha/beta hydrolase fold. Protein Eng.

[pcbi-0010028-b14] Brzozowski AM, Derewenda U, Derewenda ZS, Dodson GG, Lawson DM (1991). A model for interfacial activation in lipases from the structure of a fungal lipase-inhibitor complex. Nature.

[pcbi-0010028-b15] Nardini M, Lang DA, Liebeton K, Jaeger KE, Dijkstra BW (2000). Crystal structure of *Pseudomonas aeruginosa* lipase in the open conformation. The prototype for family I.1 of bacterial lipases. J Biol Chem.

[pcbi-0010028-b16] Fersht AR, Dagget V (2002). Protein folding and unfolding at atomic resolution. Cell.

[pcbi-0010028-b17] Warshel A (2003). Computer simulations of enzyme catalysis: Methods, progress and insights. Annu Rev Biophys Biomol Struct.

[pcbi-0010028-b18] Peters GH, Bywater RP (1999). Computational analysis of chain flexibility and fluctuations in *Rhizomucor miehei* lipase. Protein Eng.

[pcbi-0010028-b19] Jensen M, Jensen TR, Kjaer K, Bjornholm T, Mouritsen OG (2002). Orientation and conformation of a lipase at an interface studied by MD simulations. Biophys J.

[pcbi-0010028-b20] Peters GH, Bywater RP (2001). Influence of a lipd interface on protein dynamics in a fungal lipase. Biophys J.

[pcbi-0010028-b21] Ottoson J, Fransson L, Hult K (2002). Substrate entropy in enzyme enantioselectivity: An experimental and molecular modeling study of a lipase. Protein Sci.

[pcbi-0010028-b22] James JJ, Lakshmi BB, Raviprasad V, Ananth MJ, Kanguene P (2003). Insights into pH-dependent enantioselective hydrolysis of ibuprofen esters by *Candida rugosa* lipase. Protein Eng.

[pcbi-0010028-b23] Gunasekaran K, Ma B, Nussinov R (2003). Triggering loops and enzyme function: Identification of loops that trigger and modulate movements. J Mol Biol.

[pcbi-0010028-b24] Lindahl E, Hess B, van der Spoel D (2001). GROMACS 3.0: A package for molecular simulation and trajectory analysis. J Mol Mod.

[pcbi-0010028-b25] Berendsen HJC, van der Spoel D, van Drunen R (1995). GROMACS: A message-passing parallel MD implementation. Comp Phys Comm.

[pcbi-0010028-b26] Essman U, Perela L, Berkowitz ML, Darden T, Lee H (1995). A smooth particle mesh Ewald method. J Chem Phys.

[pcbi-0010028-b27] Berendsen HJC, Postma JPM, Dinola A, Haak JR (1984). MD with coupling to an external bath. J Chem Phys.

[pcbi-0010028-b28] Miyamoto S, Kollman PA (1992). SETTLE: An analytical version of the SHAKE and RATTLE algorithms for rigid water models. J Comp Chem.

[pcbi-0010028-b29] Hess B, Bekker H, Berendsen HJC, Fraaije JGEM (1997). LINCS: A linear constraint solver for molecular simulations. J Comp Chem.

[pcbi-0010028-b30] Eisenhaber F, Lijnzaad, Argos P, Sander C, Scharf M (1995). The double cube lattice method: Efficient approaches to numerical integration of surface area and volume and to dot surface contouring of molecular assemblies. J Comp Chem.

[pcbi-0010028-b31] Vriend G (1990). WHATIF: A molecular modeling and drug design program. J Mol Graph.

