# Supplementary material for: Evidence of a Double-Lid Movement in Pseudomonas aeruginosa Lipase: Insights from Molecular Dynamics Simulations
Source: PLoS Comput Biol. 2005 Aug 12;1(3):e28. doi: 10.1371/journal.pcbi.0010028 (PMC1187864; doi:10.1371/journal.pcbi.0010028)
Supplement: Supplementary file 1 [file pcbi.0010028.sd001.pdf]

Lipases

Lipases are a group of ubiquitous enzymes found in almost all organisms from bacteria to man. They belong to the hydrolase class of enzymes and can catalyze both the forward and reverse reaction of hydrolyzing an ester bond of a long chain fatty acid. There is no strict definition of “long”. Ideally any acyl ester having more than 10 carbon atoms is a right candidate for being a lipase substrate. Historically, lipases have been known from the start of the previous century (Eijkman C, Parasitenk. Infektionskr 1901 29: 841-848). But until recently these enzymes have not been understood very well. Industrially these have a high market for their use in detergents and soaps. Structurally, they have an alpha/beta hydrolase fold. (Fig. S1.1) - eight-stranded beta sheet packed by two layers of amphiphilic alpha helices. The active site is the traditional catalytic triad: Serine, Histidine and Aspartate.

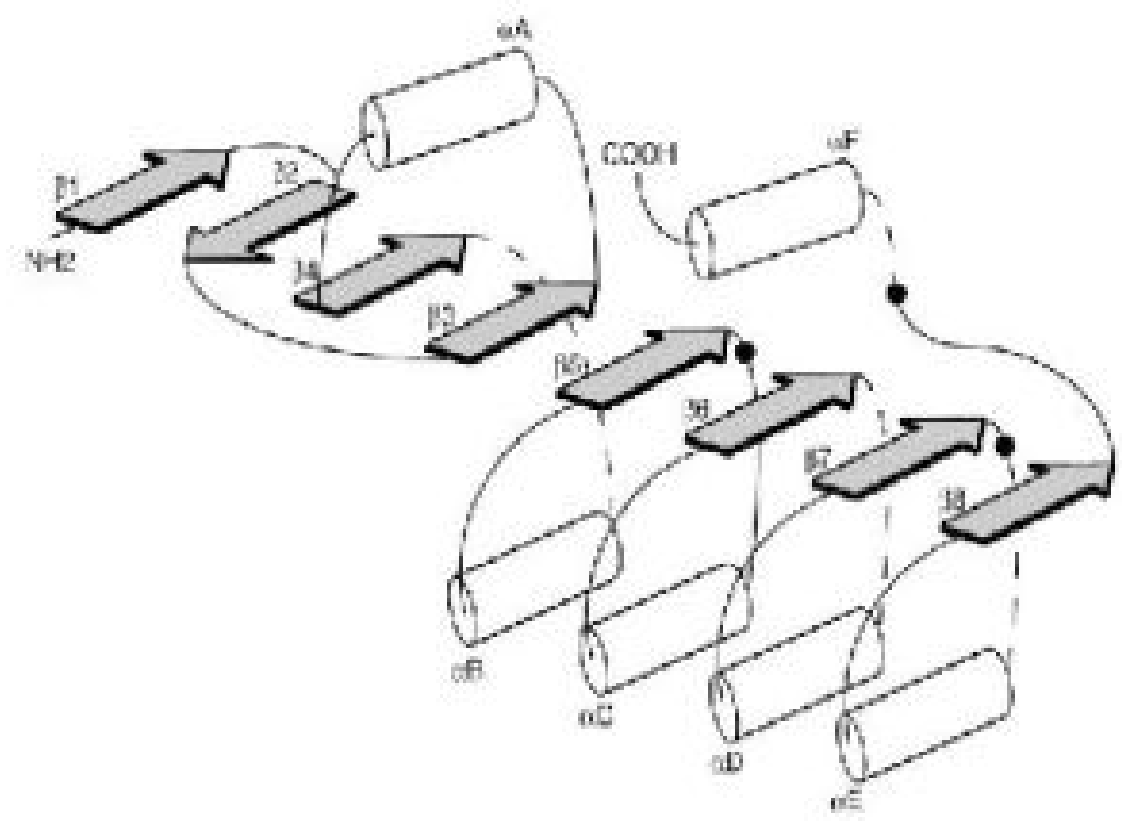

Figure S1.1 Topology diagram showing the structure of the canonical alpha/beta hydrolase fold.

An interesting factor about lipases is interfacial activation. Lipases are activated only in an oil-water interface. In 1990, two diverse lipases were crystallized, a Rhizomucor meihei lipase by Brady et al and the other, a human pancreatic lipase by Winkler and co workers (Fig S1.2). These structures revealed a unique mechanism by which this interfacial activation could be understood. A region of the protein was highly flexible and could move to cover the active pocket in an aqueous environment. When the lipase found itself in an oil-water interface, this region opens up and the active sites lie exposed.

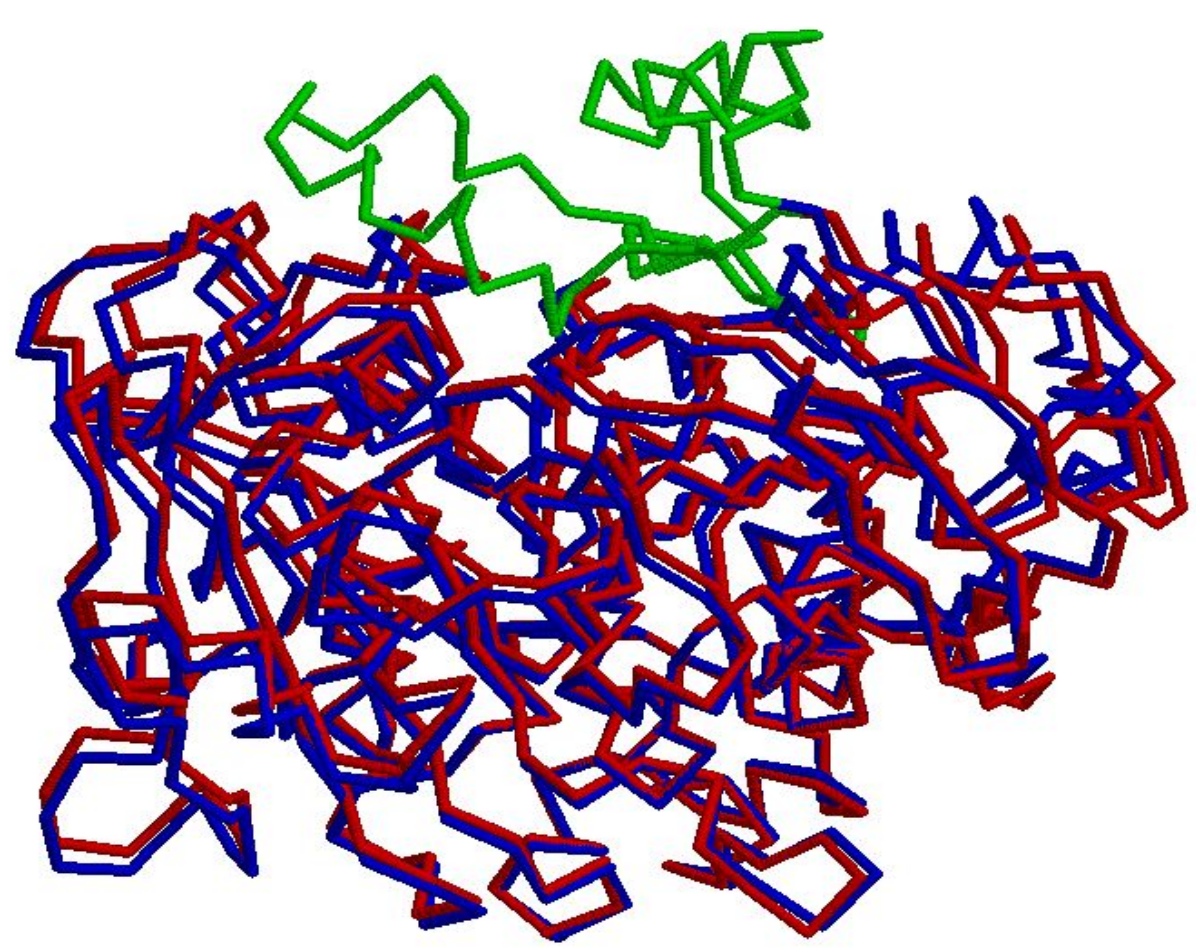

Figure S1.2 A comparison of the open and the closed forms of *Candida rugosa* lipase. The lid sub-domain is shown in green. The rest of the protein is relatively rigid, as can be seen from the superimposition of the red (open) and blue (closed) backbones.
