# Supplementary material for: Evidence of a Double-Lid Movement in Pseudomonas aeruginosa Lipase: Insights from Molecular Dynamics Simulations
Source: PLoS Comput Biol. 2005 Aug 12;1(3):e28. doi: 10.1371/journal.pcbi.0010028 (PMC1187864; doi:10.1371/journal.pcbi.0010028)
Supplement: Supplementary file 2 [file pcbi.0010028.sd002.pdf]

Molecular Dynamics Simulations: A short perspective

To analyze a system of particles at a microscopic level, computational modeling has been used successfully. The first report of such a simulation was reported by Alder and Wainwright in 1957 (Alder and Wainwright Journal Of Chemical Physics 27:1208-09) Here they describe a system where particles are defined as hard spheres and the collision between them are studied at different temperature. The first protein to be simulated was BPT1 way back in 1977 (McCammon et al Nature 1977 267:585-90). This involved studying a protein molecule in vacuum. From this we have come a long way so as to describe bigger macromolecules for long time durations and in more realistic conditions. For example in the simulation described in the paper, we use explicit water and applied periodic boundary conditions which are now commonly used. In recent years it has also become a common practice to validate structures of molecules obtained by NMR or X-ray crystallography using molecular dynamics (Hansson et al , Current Opinion in Structural Biology 2002 12:190-96). Newer forcefields have ensured that MD simulations are valid for membrane proteins also.

Another field where molecular dynamics studies are extensively used is in peptide folding simulations. Newer and better forcefield parameters are being developed in this regard. In our lab we have investigated the folding process of a beta hairpin and have shown through in silico mutation that salt bridges can effectively substitute hydrophobic interactions in folding. At present we are also investigating the folding intermediates of a Trp cage using molecular dynamics simulation (Aswin Sai Narain Seshasayee, Theoretical Biology and Medical Modelling, Accepted).

What was earlier sequence to structure to function is now sequence to structure to dynamics to functions. A lot of this has to do with MD simulations
