# Supplementary material for: Evidence of a Double-Lid Movement in Pseudomonas aeruginosa Lipase: Insights from Molecular Dynamics Simulations
Source: PLoS Comput Biol. 2005 Aug 12;1(3):e28. doi: 10.1371/journal.pcbi.0010028 (PMC1187864; doi:10.1371/journal.pcbi.0010028)

Molecular Dynamics Simulations: A Model Protocol

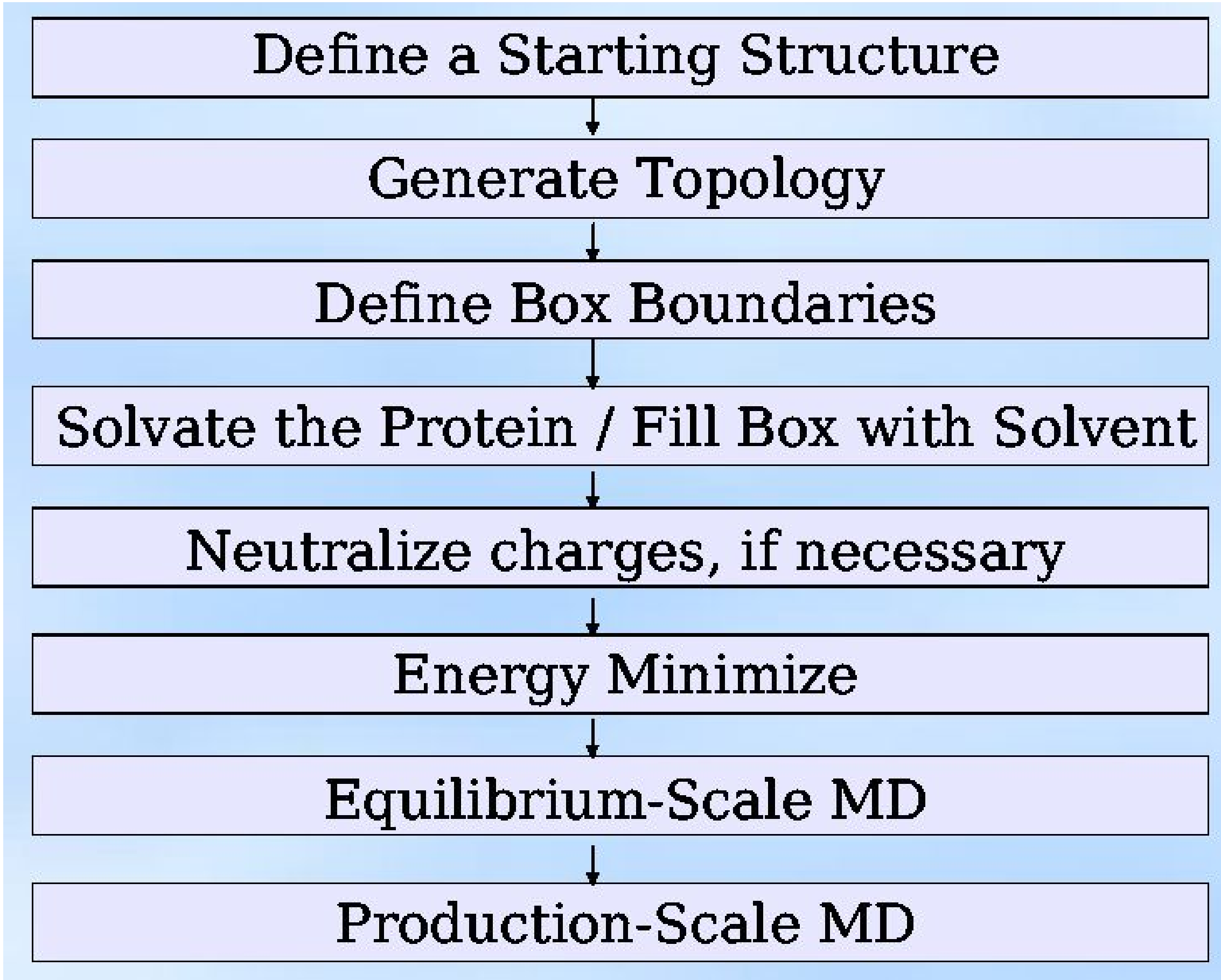

Supplement: Supplementary file 3 [file pcbi.0010028.sd003.pdf]
