# Supplementary material for: Evidence of a Double-Lid Movement in Pseudomonas aeruginosa Lipase: Insights from Molecular Dynamics Simulations
Source: PLoS Comput Biol. 2005 Aug 12;1(3):e28. doi: 10.1371/journal.pcbi.0010028 (PMC1187864; doi:10.1371/journal.pcbi.0010028)
Supplement: Supplementary file 4 [file pcbi.0010028.sd004.pdf]

Molecular Dynamics Simulations: Position Restraints

In position restraint simulations, a set of atoms are fixed in space and any departure from that fixed position is heavily penalized in the potential function. In order to investigate the interdependence of flap movement we had used position restrained molecular dynamics simulations. This is a hypothetical step and there is no proper experimental equivalent to this. The idea is that such simulations enable us to explicitly study the effect of the movement of a portion of the molecule under study on another. By restraining either of the two lids in our simulations, we have been successful in showing the extent of dependence of the movement of one lid on the other.
