# Supplementary material for: Evidence of a Double-Lid Movement in Pseudomonas aeruginosa Lipase: Insights from Molecular Dynamics Simulations
Source: PLoS Comput Biol. 2005 Aug 12;1(3):e28. doi: 10.1371/journal.pcbi.0010028 (PMC1187864; doi:10.1371/journal.pcbi.0010028)
Supplement: Supplementary file 5 [file pcbi.0010028.sd005.pdf]

Simulation Parameters

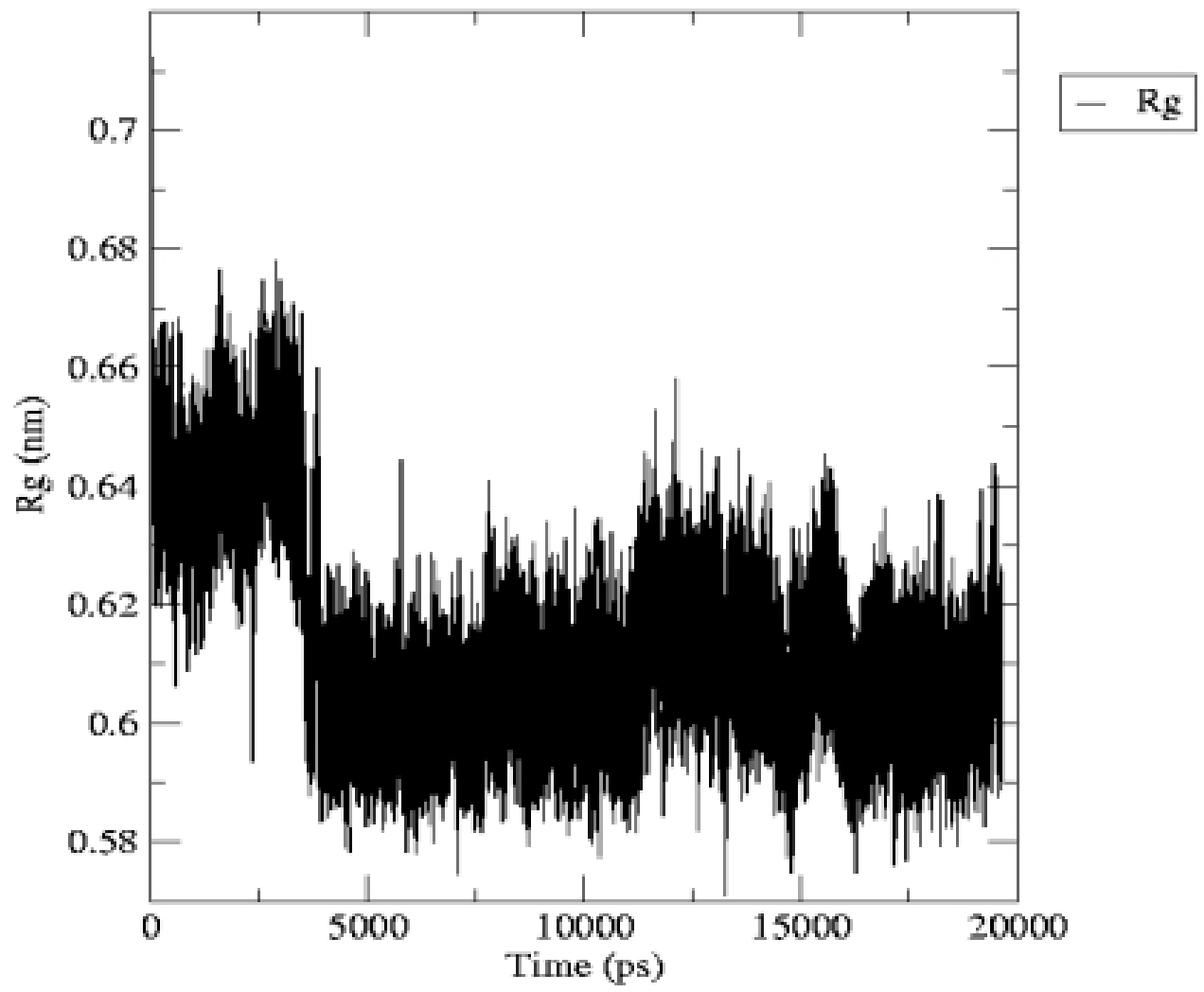

(A)

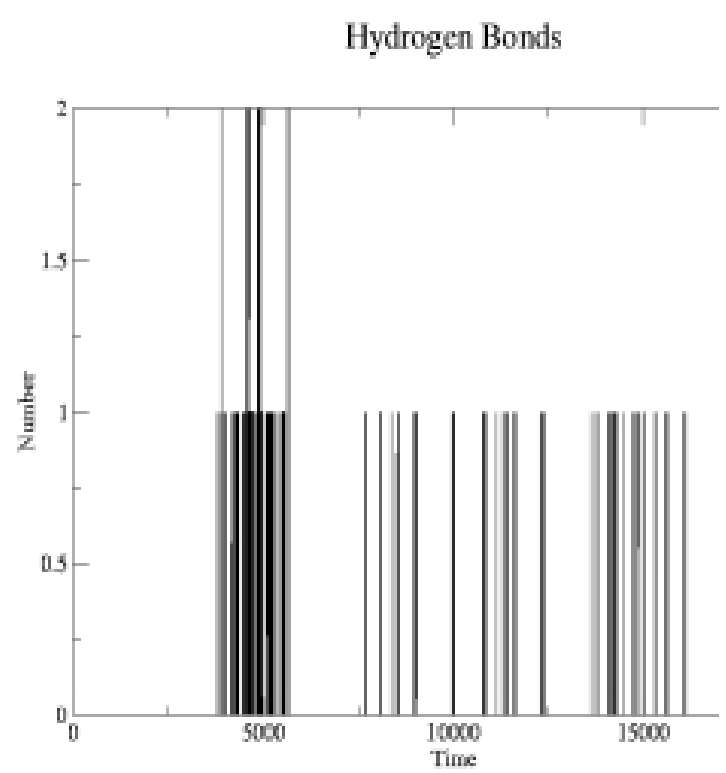

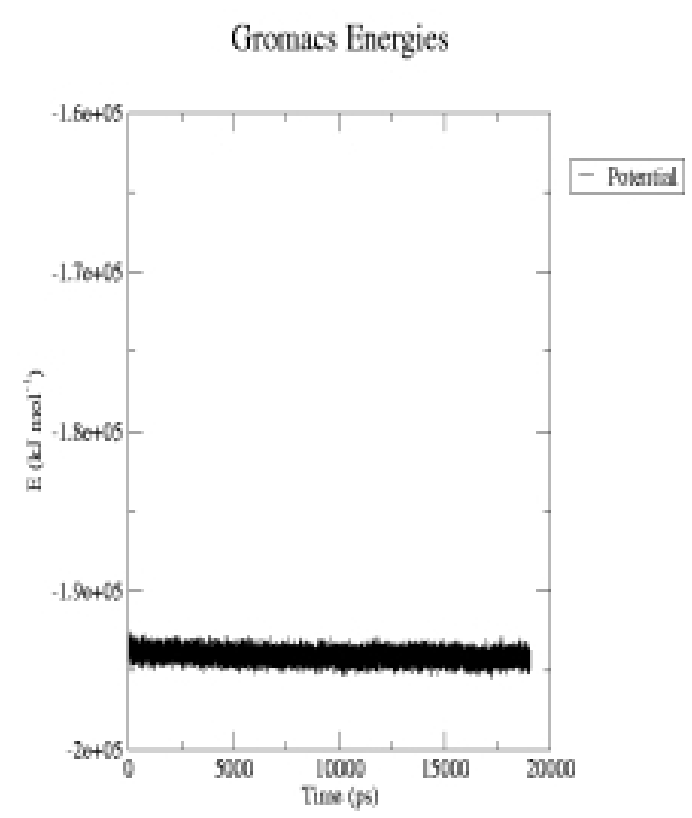

(B) (C)

(A) This shows the time variation of the radius of gyration against time. (B) We initially analyzed the two lids for the formation of hydrogen bonds so as to stabilize the closed form. But this is not so. As seen from the figure there is almost no hydrogen bond formation across the two lids. (C) The figure represents potential energy against time course of simulation. The energy was found to be constant implying equilibration.
