# Supplementary material for: Evidence of a Double-Lid Movement in Pseudomonas aeruginosa Lipase: Insights from Molecular Dynamics Simulations
Source: PLoS Comput Biol. 2005 Aug 12;1(3):e28. doi: 10.1371/journal.pcbi.0010028 (PMC1187864; doi:10.1371/journal.pcbi.0010028)
Supplement: Supplementary file 6 [file pcbi.0010028.sd006.pdf]

The Third Mobile Loop

After the simulation we noticed that there were actually three regions which showed great deviation from the starting structure. The most mobile region was lid1. In addition there were two more regions which also showed marked movement in the course of the simulation. Interestingly both were near lid1. The region 200-230 has been discussed in the paper and shown to be lid2. The third region (residues20-30) appears close to lid1 but the movement of this stretch does not really help in covering the binding pocket.Hence we have not considered this as a lid.

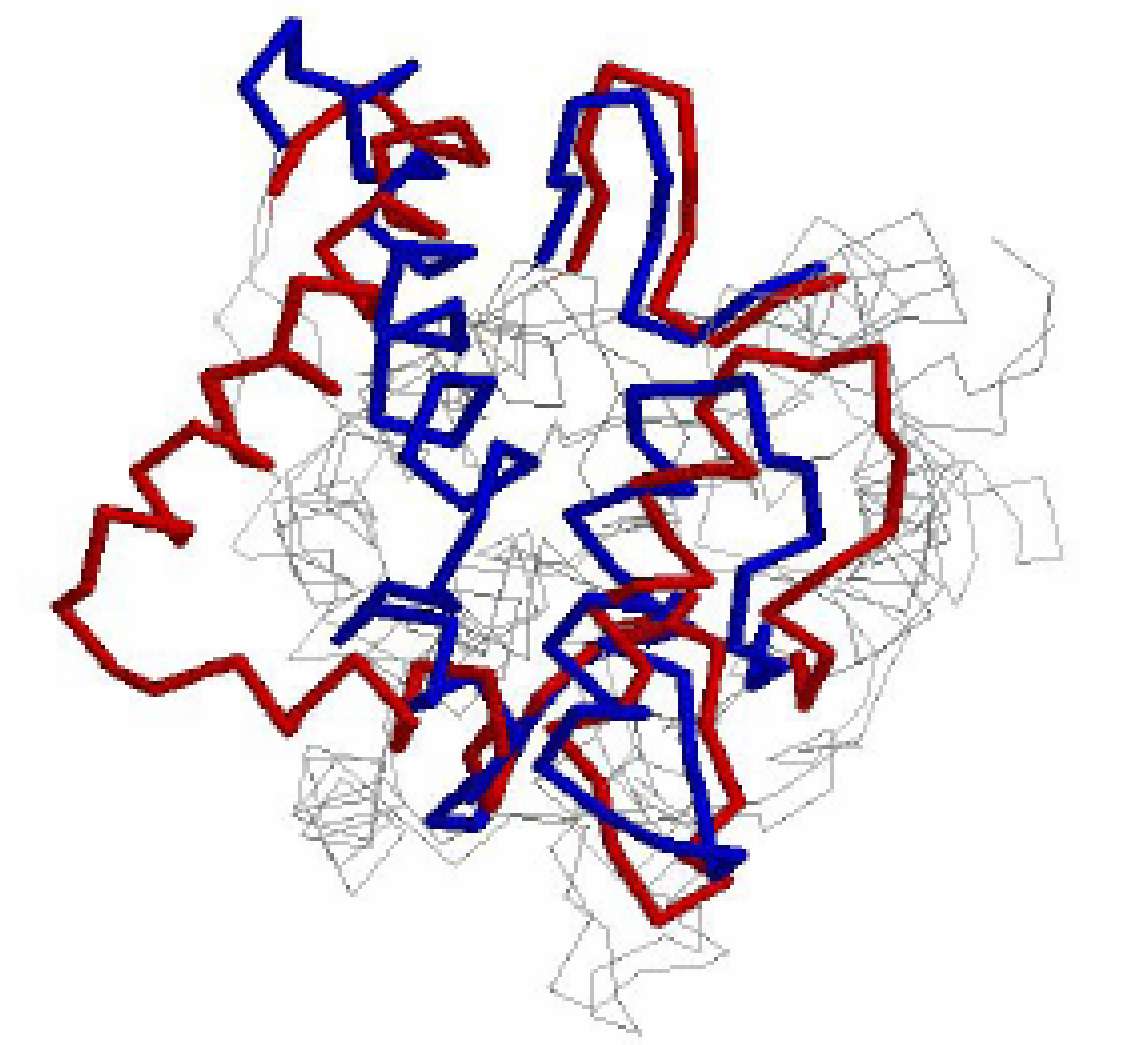

Figure S6.1 Superposition of the start structure (red) and the 10ns structure (blue). The three mobile regions are shown.

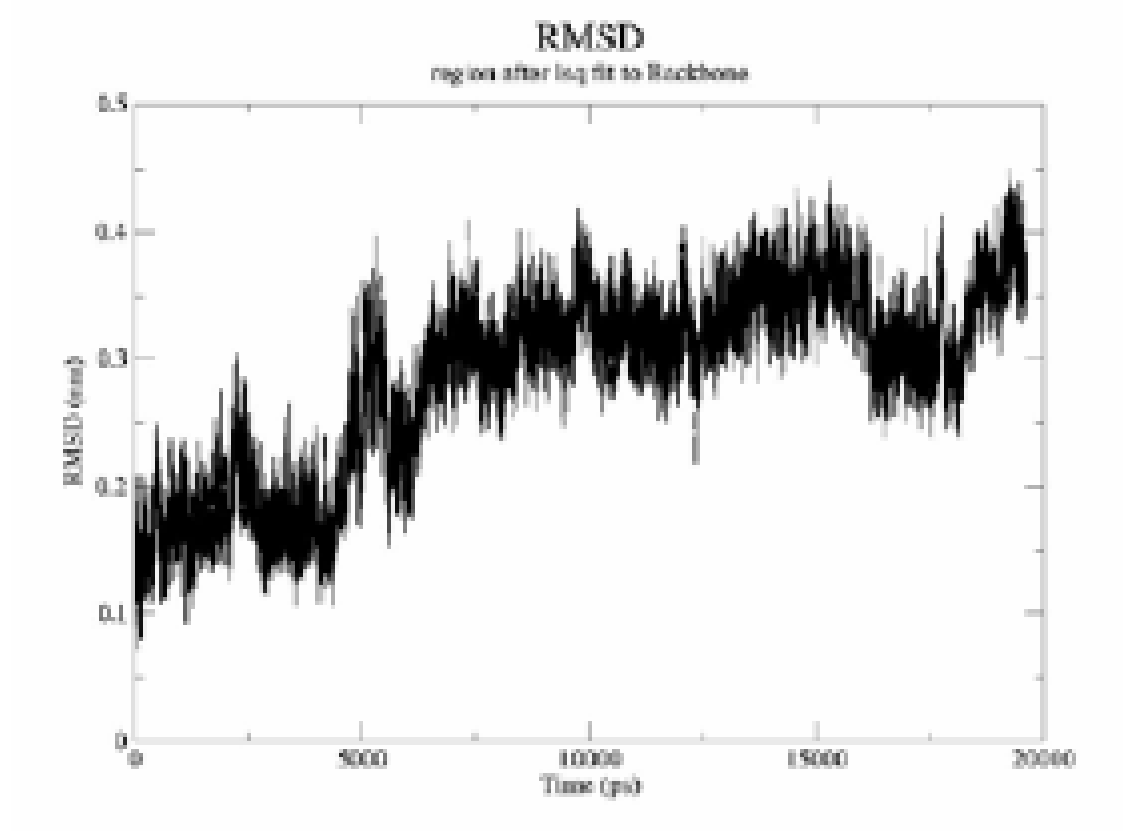

Figure S6.2 RMSD of the third mobile region: 20-30 after least squares fit of the protein backbones
