# Supplementary material for: Evidence of a Double-Lid Movement in Pseudomonas aeruginosa Lipase: Insights from Molecular Dynamics Simulations
Source: PLoS Comput Biol. 2005 Aug 12;1(3):e28. doi: 10.1371/journal.pcbi.0010028 (PMC1187864; doi:10.1371/journal.pcbi.0010028)

Single mutants

This figure shows a superimposition of the wild-type closed form and the final structure obtained from the F214S simulation. The lid residues are highlighted.

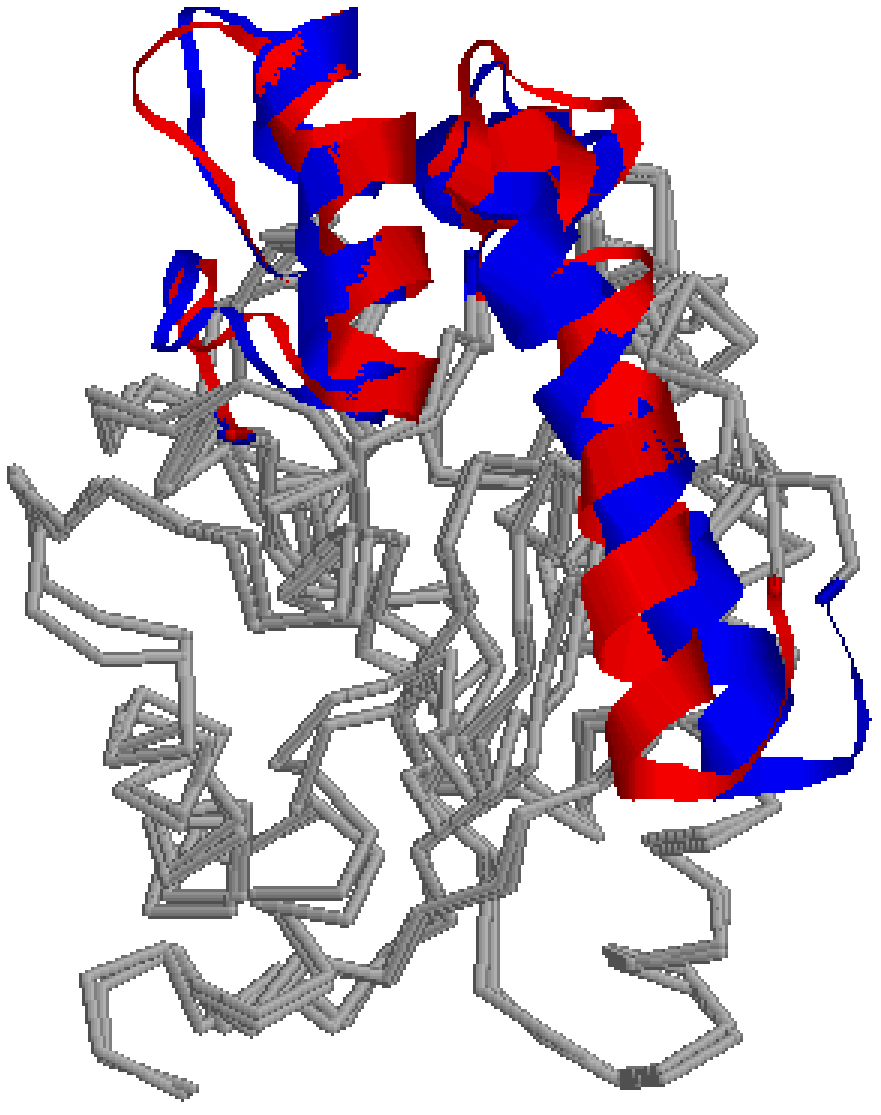

Supplement: Supplementary file 7 [file pcbi.0010028.sd007.pdf]
