# Supplementary material for: Evidence of a Double-Lid Movement in Pseudomonas aeruginosa Lipase: Insights from Molecular Dynamics Simulations
Source: PLoS Comput Biol. 2005 Aug 12;1(3):e28. doi: 10.1371/journal.pcbi.0010028 (PMC1187864; doi:10.1371/journal.pcbi.0010028)
Supplement: Supplementary file 8 [file pcbi.0010028.sd008.pdf]

Box Sizes

**Water Box**	*5.511 x 4.933 x 4.964*
**Water+Octane box**	*5.511 x 4.933 x 7.5*
